# [Retracted] MicroRNA‑140 represses glioma growth and metastasis by directly targeting ADAM9

**DOI:** 10.3892/or.2024.8723

**Published:** 2024-03-06

**Authors:** Xiaogang Liu, Shanjun Wang, Aiqin Yuan, Xunhui Yuan, Bing Liu

Oncol Rep 36: 2329–2338, 2016; DOI: 10.3892/or.2016.5007

Following the publication of this paper, it was drawn to the Editor's attention by a concerned reader that certain of the Transwell cell invasion and migration assay data shown in Figs. 2C and 5D were strikingly similar to data in different form in other articles written by different authors at different research institutes, which had either already been published or had been submitted for publication at around the same time (some of which have now been retracted).

Owing to the fact that certain of the data in the above article had already been published prior to its submission to *Oncology Reports*, the Editor has decided that this paper should be retracted from the Journal. The authors were asked for an explanation to account for these concerns, but the Editorial Office did not receive a reply. The Editor apologizes to the readership for any inconvenience caused.

